# Supplementary material for: Prevalence and associated factors of pregnancy induced hypertension among pregnant women in public hospitals of Hadiya Zone, Central Ethiopia: A cross-sectional study
Source: PLoS One. 2025 Aug 1;20(8):e0326236. doi: 10.1371/journal.pone.0326236 (PMC12316221; doi:10.1371/journal.pone.0326236)
Supplement: Attachment — Submitted filename: PIH comment.pdf [file pone.0326236.s002.pdf]

Dear Authors,

Thank you for your valuable contribution to the literature on pregnancy-induced hypertension (PIH). Your study, "Prevalence and Associated Factors of Pregnancy Induced Hypertension among Pregnant Women in Public Hospitals of Hadiya zone, Southern Ethiopia: A cross-sectional study," addresses a critical public health issue and provides important insights into the risk factors for PIH in this region.

Your findings highlight the significant burden of PIH in Hadiya zone and underscore the need for targeted interventions to improve maternal health outcomes. The identification of key risk factors, such as wealth index, gestational age, overweight/obesity and family history of hypertension, can help healthcare providers to identify high-risk women and implement timely preventive measures.

While your study has made a valuable contribution, I would like to offer a few suggestions for improvement:

- A Systematic Review and Meta-Analysis on PIH in Ethiopia Already Exists. This is a valid concern. While your study provides valuable insights into the local context of Hadiya zone, it's crucial to differentiate your contribution from existing systematic reviews and meta-analyses. By focusing on these aspects, you can add value to the existing body of knowledge and provide actionable recommendations for improving maternal health outcomes. So, authors should be incorporate these suggestion in back ground of the study to strengthen your study.
- In study setting and design – "On average, the total number of mothers serviced by all hospitals in 2011 was 10,774. The data provided is outdated (2011) and may not reflect the current situation. It is recommended to use more recent data for accurate information.
- Source and study population

  "The study population consisted of consented pregnant women who visited ANC follow-up in the selected hospitals of the Hadiya zone, and whose gestational age was greater than 20 weeks during the study period". It's confused me. What mean the phrase "consisted of consented pregnant women?"

- Mothers who have a previous history of hypertension were excluded from the study. Why? do you have justification
- Sampling procedure section—last sentences "pregnant women attending ANC in the respective hospital were included consecutively until the desired sample size was reached'. The authors chose to use consecutive sampling, which involves including all eligible participants who meet the inclusion criteria until the desired sample size is reached. Why not sampled? And also, not acknowledge the potential for selection bias and limited generalizability in the discussion section.
- Data collection tools and procedures
    - o The sentences... Data on proteinuria and other clinical variables was taken from the medical charts of pregnant women. However, is proteinuria data, a critical factor in classifying different types of pregnancy-induced hypertension (PIH), readily accessible from the medical charts of all pregnant women? This information allowed for accurate categorization of PIH cases based on established diagnostic criteria. so, Why was not taken a sample directly from women?
    - o Measurement of blood pressure was taken by using standard procedures, with a digital sphygmomanometer. Better if more elaborate the standard procedure for measuring blood pressure using a digital sphygmomanometer for the general audience easily understanding
- Data Processing and Analysis
    - o Those variables with a P value ≤0.25 in the bivariable analysis... what is your baseline reference to take a P value ≤0.25? Why not take 0.2, 0.3, 0.4, etc.? Do you have any justification?
- Study variable
    - o Obstertric variable—the variable "mode of delivery'- it is the previous or current mode of delivery? Additionally, I did not see any figures about this variable in result sections.
- Operational definitions
    - o There was no real definition of PIH used. "Pregnancy-induced hypertension (PIH) "is systolic blood pressure ≥ 140 mm Hg or diastolic blood pressure ≥ 90 mmHg after gestation age of twenty weeks in a previously average hypertensive woman".

This definition contradicts the definition written in 1st paragraph of your background, "Pregnancy-induced hypertension (PIH) "is systolic blood pressure ≥ 140 mm Hg or diastolic blood pressure ≥ 90 mmHg after gestation age of twenty weeks in a previously normal BP". It would be important to define clearly and should be referenced where it take the definition.

- Better if define wealth index
- Elaborate how to measure the BMI
- Author's defined HELLP Syndrome: It is a variant of preeclampsia characterized by hemolysis (H), elevated liver enzymes (EL), and low platelet count (LP). But I did see any where those are written in manuscript. So, what importance this variable, better if removed.

Result

- Grammar error….Most participants (429, 99.1%) were married. Edit as Most participants (99.1%) were married
- In multivariate of factors associated with PIH; the CI of variable family of history DM is wider (7.67(2.22-26.453)). Why? do you have justification

Discussion

- Authors compared the finding of prevalence of PIH within Ethiopia only. Better if additionally compared with Sub-Saharan Africa, globally.
- Again authors said… The discrepancy in the findings could be attributed to the differences in settings, study period, and socio-economic characteristics of the pregnant women. While the all studies were conducted in Ethiopia, there could be variations in specific methodologies, sample sizes, and data collection techniques that might influence the findings rather than study setting. Additionally, subtle differences in healthcare access, cultural practices, and dietary habits within different regions of Ethiopia could contribute to variations in PIH prevalence and associated factors. It's important to critically evaluate the methods and limitations of each study to identify potential sources of discrepancy. By carefully considering these factors, researchers can better understand

the nuances of PIH in different Ethiopian contexts and develop more targeted prevention and management strategies.

- Discussion section para 2 seems to be the conclusion. I recommend the authors combine the second paragraph of the discussion section with the conclusion section to improve the flow and clarity of the manuscript
