# Supplementary material for: Prevalence and associated factors of pregnancy induced hypertension among pregnant women in public hospitals of Hadiya Zone, Central Ethiopia: A cross-sectional study
Source: PLoS One. 2025 Aug 1;20(8):e0326236. doi: 10.1371/journal.pone.0326236 (PMC12316221; doi:10.1371/journal.pone.0326236)
Supplement: Attachment — Submitted filename: Point by point response 2.docx [file pone.0326236.s005.docx]

**Point-to-point responses**

Dear Adera Debella Kebede, MSC
Academic Editor
PLOS ONE

Thank you for the opportunity to review our manuscript. We thank you and the reviewers for the insightful comments that have improved our manuscript. We have addressed all the comments and submitted the revised version of the manuscript to the online portal. Please kindly receive our point-by-point responses to the comments as follows:

6. Review Comments to the Author

Please use the space provided to explain your answers to the questions above. You may also include additional comments for the author, including concerns about dual publication, research ethics, or publication ethics. (Please upload your review as an attachment if it exceeds 20,000 characters)

Reviewer #1: Dear authors, Thanks for addressing all my comments. I recommend you 1. Please ensure that your manuscript meets PLOS ONE's style requirements. 2. Please move your ethical approval statement to the end of methods sections. 3. Authors should correct in manuscript some typographical and grammatical errors before publication.

**Response to Reviewer 1 comments**

1. Please ensure that your manuscript meets PLOS ONE's style requirements.

**Response: We have formatted as per the PLOS ONE's style requirements.**

**2.** Please move your ethical approval statement to the end of methods sections.

**Response: We moved the ethical approval statement to the end of the methods**

**section**.

3.Authors should correct in manuscript some typographical and grammatical errors before

publication.

**Response: We corrected some typographical and grammatical errors in the**

**manuscript.**

Reviewer #2: All comments have been addressed. except, URL is not added in reference numbers 3 and 7 in the revised manuscript.

**Response to Reviewer 2 comments**

**Response:** We have added the URLs for reference numbers 3 and 7 in the revised

manuscript.

7. PLOS authors have the option to publish the peer review history of their article ([what does this mean?](https://journals.plos.org/plosone/s/editorial-and-peer-review-process#loc-peer-review-history)). If published, this will include your full peer review and any attached files.


If you choose “no”, your identity will remain anonymous but your review may still be made public.


**Do you want your identity to be public for this peer review?** For information about this choice, including consent withdrawal, please see our [Privacy Policy](https://www.plos.org/privacy-policy).

Reviewer #1: **Yes:** Ebisa Zerihun

Reviewer #2: No

**Response: No**
